# Supplementary material for: Dispersive-wave induced noise limits in miniature soliton microwave sources
Source: Nat Commun. 2021 Mar 4;12:1442. doi: 10.1038/s41467-021-21658-7 (PMC7933157; doi:10.1038/s41467-021-21658-7)
Supplement: Supplementary file 1 — Supplementary Information [file 41467_2021_21658_MOESM1_ESM.pdf]

Supplementary Information to Dispersive-wave induced noise limits in miniature soliton microwave sources

Qi-Fan Yang[1,*], Qing-Xin Ji[1,*], Lue Wu[1,*], Boqiang Shen[1], Heming Wang[1], Chengying Bao[1], Zhiquan Yuan[1], and Kerry Vahala[1,†]
[1]T. J. Watson Laboratory of Applied Physics, California Institute of Technology, Pasadena, California 91125, USA
*These authors contributed equally to this work.
†Corresponding author: vahala@caltech.edu

I. COMPARISON BETWEEN ANALYTICAL AND NUMERICAL RESULTS

Under the approximation $|\kappa_{\rm S} - \kappa_{\rm D}| \ll G, \Delta\omega$; and also taking $\Delta\omega$ and G as comparable, the lower branch of the hybridized mode reads,

$$\omega_{r-} = \frac{\omega_{r\rm S} + \omega_{r\rm D}}{2} - \sqrt{G^2 + \frac{\Delta\omega^2}{4}}, \tag{S1}$$

$$h_{r-} = \frac{G a_r + \Delta\omega' b_r}{\sqrt{\Delta\omega'^2 + G^2}}, \tag{S2}$$

where $\Delta\omega$ is the frequency difference between the two non-hybridized mode, and $\Delta\omega' = \Delta\omega/2 + \sqrt{\Delta\omega^2/4 + G^2}$ is the frequency difference between the hybridized lower branch h_{r-} and the non-hybridized soliton mode.

Numerical simulations based on coupled Lugiato-Lefever equations (LLE) are performed to verify the results obtained using the analytical model. Note that the coupling between the soliton mode and dispersive wave mode leads to mode hybridization, while in LLE equations non-hybridized mode frequencies should be used. The mode family

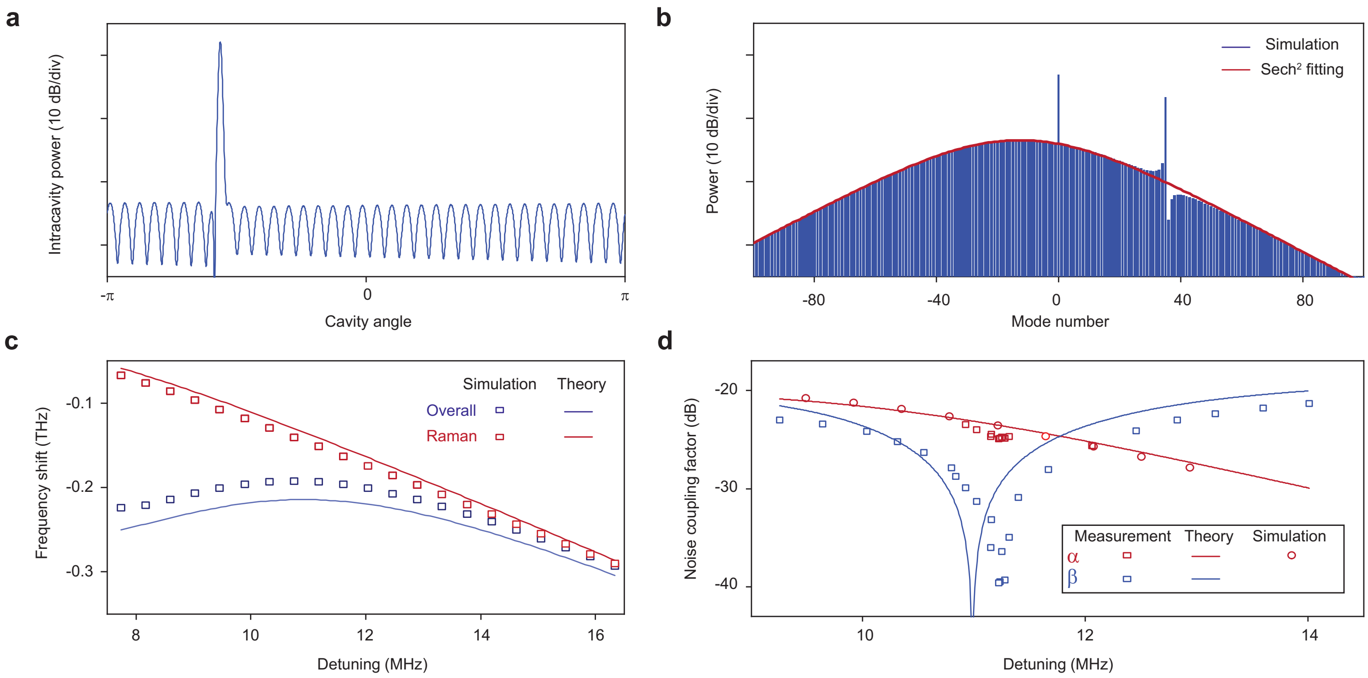

Fig. S1. **Simulations of soliton dynamics and noise transduction factors.** **a,** Simulated intracavity field of a soliton with a strong single-mode dispersive wave. **b,** The optical spectrum of the soliton in panel a. 2048 modes participated in the simulation. The red line denotes sech^2 fitting of the spectral envelope. **c,** Comparison between analytical and simulated results of frequency shifts. **d,** Simulated α factor versus analytical results.

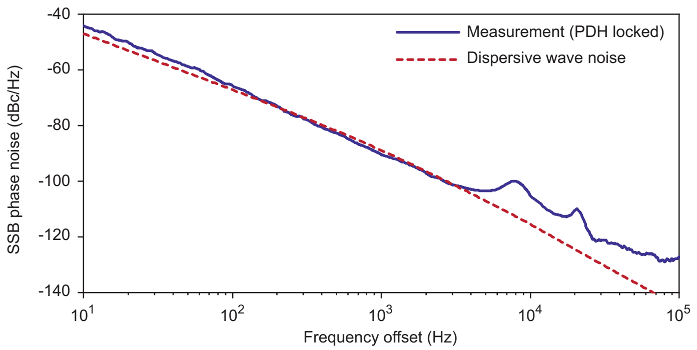

Fig. S2. **Phase noise limit using PDH locking technique.** Measured phase noise limit using Pound-Drever-Hall locking technique is plotted along with the predicted dispersive wave noise limit.

dispersion of non-hybrid modes used in the simulation can be approximated as

$$\omega_{\mu\mathrm{S,D}} = \omega_{\mathrm{oS,D}} + \mu D_{1\mathrm{S,D}} + \frac{1}{2}\mu^2 D_{2\mathrm{S,D}} + \mathcal{O}(\mu^3). \tag{S3}$$

At mode number $\mu = 0$, the two modes differ by 1.5247 GHz in frequency. All parameters are identical with those used in the analytical model.

A representative power distribution of the intracavity field obtained from simulation is plotted in Fig. S1a versus the 2π round-trip angle of the cavity. It features a pulse with an oscillating tail in the azimuthal frame[1]. The corresponding spectrum is plotted in Fig. S1b, and shows a sech^2 envelope and a strong dispersive-wave spike at the 35th mode. The simulated frequency shift of the soliton versus laser-cavity detuning is plotted in Fig. S1c, and exhibits excellent agreement with the analytical model. The thermal noise transduction factor (α) is also evaluated numerically by perturbing $\Delta\omega$ and calculating the changing of repetition rate, and is consistent with the analytical model as shown in Fig. S1d.

II. ADDITIONAL MEASUREMENTS

Phase noise of soliton repetition rate is also measured with Pound–Drever–Hall locking technique. The minimal phase noise is plotted in Fig. S2, along with the dispersive wave noise limit. Under these conditions, the pump frequency tracks the cavity resonance thereby suppressing its technical noise contribution to the soliton phase noise. As expected the measured noise spectrum shows a limitation consistent with the dispersive wave noise limit.

[1] Yi, X. *et al.* Single-mode dispersive waves and soliton microcomb dynamics. *Nat. Commun.* **8**, 14869 (2017).
